# Plasma polyunsaturated fatty acid concentrations and sleep apnea risk: A two-sample Mendelian randomization study

**DOI:** 10.3389/fnut.2022.956900

**Published:** 2022-08-18

**Authors:** Jiao Wang, Yingyue Huang, Huiling Yang, Zihong Lin, Adrian I. Campos, Miguel E. Rentería, Lin Xu

**Affiliations:** ^1^School of Public Health, Sun Yat-sen University, Guangzhou, China; ^2^Eastern-Fusion Master Studio of Hezhou, Hezhou, China; ^3^Hezhou Research Institute of Longevity Health Science, Hezhou, China; ^4^Department of Genetics & Computational Biology, QIMR Berghofer Medical Research Institute, Herston, QLD, Australia; ^5^Li Ka Shing Faculty of Medicine, School of Public Health, The University of Hong Kong, Hong Kong, Hong Kong SAR, China

**Keywords:** plasma polyunsaturated fatty acid, omega-3, omega-6, sleep apnea, Mendelian randomization

## Abstract

**Background:**

Previous observational studies have found that lower levels of circulating polyunsaturated fatty acids (PUFAs) were associated with a higher risk of sleep apnea (SA). However, the causality of the association remains unclear.

**Materials and methods:**

We used the two-sample Mendelian randomization (MR) study to assess the causal association of omega-3 and omega-6 fatty acids with SA. Single-nucleotide polymorphisms (SNPs) predicting the plasma level of PUFAs at the suggestive genome-wide significance level (*p* < 5 × 10^–6^) were selected as instrumental variables (IVs) from the Cohorts for Heart and Aging Research in Genomic Epidemiology (CHARGE) (*n* = ∼8,000) Consortium. For outcomes, the summary-level statistics of SA were obtained from the latest genome-wide association study (GWAS), which combined five cohorts with a total number of 25,008 SA cases and 172,050 snoring cases (total = 523,366).

**Results:**

We found no association of α-linolenic acid (ALA) [odds ratio (OR) = 1.09 per% changed, 95% confidence interval (CI) 0.67–1.78], eicosapentaenoic acid (EPA) (OR = 0.94, 95% CI 0.88–1.01), docosapentaenoic acid (DPA) (OR = 0.95, 95% CI 0.88–1.02), and docosahexaenoic acid (DHA) (OR = 0.99, 95% CI 0.96–1.02) with the risk of SA using inverse-variance weighted (IVW) method. Moreover, for omega-6 PUFAs, no association between linoleic acid (LA) (OR = 0.98, 95% CI 0.96–1.01), arachidonic acid (AA) (1.00, 95% CI 0.99–1.01), and adrenic acid (AdrA) (0.93, 95% CI 0.71–1.21) with the risk of SA was found. Similarly, no associations of PUFAs with SA were found in single-locus MR analysis.

**Conclusion:**

In the current study, we first found that there is no genetic evidence to support the causal role of omega-3 and omega-6 PUFAs in the risk of SA. From a public health perspective, our findings refute the notion that consumption of foods rich in PUFAs or the use of PUFAs supplementation can reduce the risk of SA.

## Introduction

Sleep apnea (SA), a common form of sleep-disordered breathing, is characterized by brief interruptions of breathing during sleep. SA affects almost one billion adults aged 30–69 years worldwide (1) and is linked to a higher risk of cardiovascular diseases (CVDs) (2), type 2 diabetes (3), and Alzheimer’s disease (4). The development of SA involved a higher inflammatory response (5, 6). Therefore, apart from continuous positive airway pressure (CPAP), nutritional supplementation may be a possible alternative approach to decrease the risk of SA, i.e., supplementation with polyunsaturated fatty acids (PUFAs).

As the key components of cellular and intracellular membranes, PUFAs can be classified into omega-3 PUFAs and omega-6 PUFAs. Previous studies showed that PUFAs were associated with lower risks of CVDs (7), type 2 diabetes (8), and autoimmune disorders (9). PUFAs mainly include the plant-derived α-linolenic acid (ALA) and linoleic acid (LA), and seafood-derived eicosapentaenoic acid (EPA) and docosahexaenoic acid (DHA), which were associated with lower immune response (10, 11). Previous epidemiological studies showed that lower levels of circulating omega-3 PUFA were associated with a higher risk of SA (12, 13), suggesting that diet-sourced omega-3 PUFAs may be modifiable targets for SA prevention. Based on these findings and the tolerability and safety of PUFAs (14), it has been speculated that PUFAs supplementation may reduce inflammatory response and decrease the risk of SA, and intervention trials have been suggested (15).

In this situation, in which a rationale exists for studying the role of PUFAs in SA but the evidence is lacking, Mendelian randomization (MR) provides an alternative way of examining causal effects. MR is an instrumental variable (IV) approach that uses genetic variants allocated randomly at conception as IVs and thus is unlikely to be biased by common confounders, such as lifestyle, health status, and socioeconomic positions. Moreover, compared with randomized control trials (RCTs) which estimate effects during a short term, MR tends to reflect the effects of lifelong exposure to PUFAs (16). Previous MR studies showed that higher genetically predicted ALA and LA, and lower EPA and docosapentaenoic acid (DPA), were associated with a lower risk of type 2 diabetes (17). A higher genetically predicted LA was also associated with a lower risk of asthma (9). However, to date we found no MR studies regarding the association between PUFAs and SA. Using genetic variants [i.e., single nucleotide polymorphisms (SNPs)] from the genome-wide association study (GWAS) of PUFAs (18–20), we conducted two-sample MR studies to examine the effect of main omega-3 fatty acids (ALA, EPA, DPA, and DHA) and omega-6 fatty acids [LA, arachidonic acid (AA), and adrenic acid (AdrA)] on SA, using genetic summary statistics from a large multivariate genome-wide association study (GWAS) of SA.

## Materials and methods

### Study design and data sources

**FIGURE 1:**
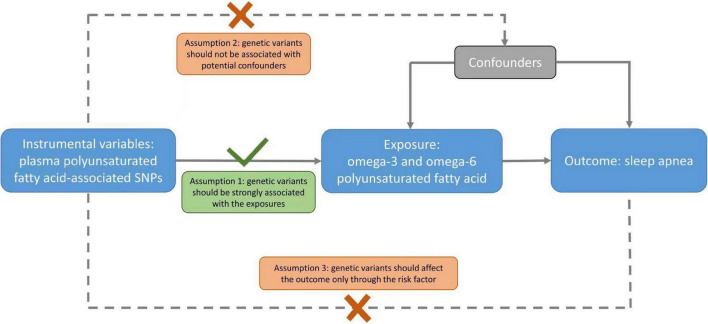
A schematic diagram of the Mendelian randomization (MR) assumptions underpinning an MR analysis of the association of plasma fatty acid levels with sleep apnea (SA). The dashed lines represent the possible violations of the MR assumptions.

We did a two-sample MR study, using de-identified summary-level data that were publicly available. Information about the data sources and sample sizes used in this study are summarized in the appendix (Supplementary Table 1). An overview of the study design is displayed in Figure 1. Ethical approvals were obtained in all original studies.

### Genetic associations with polyunsaturated fatty acids (exposure)

Genetic variants associated with plasma omega-3 and omega-6 PUFAs were obtained from the recent GWAS of plasma fatty acid in European ancestry. Single nucleotide polymorphisms (SNPs) that reach the suggestive significant genome-wide association level (*p* ≤ 5 × 10^–6^) and had a minor allele frequency of 0.01 or more were included. In this MR study, four omega-3 (ALA, EPA, DPA, and DHA) and three omega-6 (LA, AA, and AdrA) PUFAs were included. The GWAS of omega-3 (20) and omega-6 (18) PUFAs were from the Cohorts for Heart and Aging Research in Genomic Epidemiology (CHARGE) Consortium (*n* = 8,866 and 8,631). The unit of omega-3 and omega-6 was the percentage (%) of total fatty acids. Information about DHA and LA-related SNPs was obtained from an up-to-date meta-analysis of GWAS (19).

To date, the best-characterized gene loci for PUFAs are the fatty acid desaturase (FADS) genes, such as FADS1, FADS2, and FADS3. These biologically relevant candidate genes encode the δ-5 and δ-6 desaturases, which are involved in the metabolic conversion of the LA to longer chain omega-6 PUFAs. The SNPs strongly associated with fatty acid were mainly allocated in chromosome 11q12.2 (i.e., in genes of C11orf9/10, FEN1, and FADS), and chromosome 6p24.2 (i.e., ELOVL2). From the meta-analysis of GWAS, the most highly associated SNPs on chromosome 11 explained 3.8% of the variance of ALA, 2.0% of the variance of EPA, and 8.6% of the variance of DPA (20). We further conducted the mechanistically informative analysis using the strongest SNP related to fatty acid biosynthesis in chromosome 11 or 6 for each PUFA, respectively, to minimize the pleiotropic effect (single-locus MR analysis), which method has been widely used in previous studies (17, 21).

### Genetic associations with sleep apnea and snoring (outcome)

The genetic associations with SA were obtained from the most recent and largest GWAS of SA and snoring by Campos et al. (22), which used multi-trait analysis of GWAS (MTAG) (23) to boost statistical power, leveraging the high genetic correlations between SA and snoring. The SA multi-trait discovery GWAS combined five cohorts from the United Kingdom (UK Biobank; UKB), Canada (Canadian Longitudinal Study of Aging; CLSA), Australia (Australian Genetics of Depression Study; AGDS), the United States (Partner’s Healthcare Biobank), and Finland genetic research (FinnGen) with a total number of 25,008 SA cases and 172,050 snoring cases (total = 523,366) with replication in an independent sample from 23andMe (total = 1,477,352 and cases = 175,522). Totally, 43 SNPs for SA explained 0.87% variance. For each cohort, SA was coded using either International Classification of Diseases Tenth Revision (ICD-10) codes *via* primary care records or self-reported diagnostic items *via* questionnaires (Supplementary material). All cohorts were restricted to European descent individuals with the adjustment for age, sex, batch (where relevant), and genetic ancestry principal components derived from genotype data [individual cohort details have been reported elsewhere (22)]. Since this GWAS paper is currently a pre-print, we used the latest published GWAS for sleep apnea from the FinnGen Study (24), and then repeated the analysis to validate our findings in the sensitivity analysis.

### Statistical analysis

We estimated the F-statistic for each SNP as the square of the SNP-exposure association divided by the variance of the SNP-exposure association (25), and we generated the mean F-statistic for exposure (26). Independent variants (*r*^2^ < 0.01) were selected using the “clump_data” function (EUR population) of the “MR-Base” R package. In the sensitivity analysis, we replicated the MR analysis using SNPs with a *p*-value < 5 × 10^–8^. We obtained MR estimates by meta-analyzing the SNP-specific Wald estimates (effect of SNP on outcome divided by SNP on exposure) using inverse-variance weighted (IVW) with multiplicative random effects, which assumes balanced pleiotropy. The presence of heterogeneity due to pleiotropy was indicated by high Cochran’s *Q* and *I*^2^ statistics. To ensure the same effect allele was used for exposure and outcome for palindromic SNPs (coded A/T or C/G), we aligned them on effect allele frequency and the coding (forward or reverse). In sensitivity analyses, we obtained MR estimates using different methods with different assumptions, including the weighted median (WM) and MR-PRESSO. The WM (of SNP-specific Wald estimates) gives robust estimates if more than 50% of the information is derived from valid SNPs. Mendelian randomization pleiotropy residual sum and outlier (MR-PRESSO) is another way to identify horizontal pleiotropic outliers, and corrects for pleiotropy *via* outlier removal, if necessary (27). We obtained the empirical *p*-value for the MR-PRESSO global test *via* 10,000 simulations and used the outlier corrected estimate if outliers were found. The MR-Egger intercept test is used to statistically check the presence of horizontal pleiotropy. Since body mass index (BMI) could be a confounder in the associations between PUFAs and SA, multivariable MR (MVMR) was conducted to assess the associations of PUFAs with SA after adjustment for BMI (28). To adjust for multiple comparisons, the Bonferroni multiple testing correction was applied, and two-sided *p*-values of < 0.007 (0.05/7) were considered significant. Power was estimated using the approximation that the sample size for IV analysis to obtain a given power is the sample size for exposure on outcome divided by the *r*^2^ for an instrument on exposure (29). The post-power calculation was based on the results of an online tool using several parameters, such as sample size of the outcome GWAS, variance explained by selected SNPs, and expected effect size.^1^

All statistical analyses were conducted in Stata version 13.1 (StataCorp LP, College Station, TX, United States) and R version 3.6.3 (R Foundation for Statistical Computing, Vienna, Austria) using the “TwoSampleMR,” “MendelianRandomization,” and “MRPRESSO” packages. The current MR study used publicly available summary data and does not require specific ethical approval.

## Results

Totally, 6, 17, 12, and 12 SNPs associated with ALA, EPA, DPA, and DHA, respectively, were obtained at suggestive genome-wide significance (*p*-value < 5 × 10^–6^) after excluding linkage disequilibrium (*r*^2^ < 0.01) with the average *F*-statistic range from 34 to 95. Similarly, 23, 10, and 5 SNPs associated with LA, AA, and AdrA, respectively, were identified, with the average *F*-statistic range from 41 to 145. Proxy SNPs were used when there were missing SNPs in the outcome dataset. No SNP was palindromic. Supplementary Tables 2, 3 summarize the information extracted for each SA-related SNP in the current study. Using the single-locus MR analysis, only the strongest SNP associated with fatty acid in chromosomes 11 or 6 were collected to predict the levels of PUFAs, with the average *F*-statistic range from 104 to 699. Rs174547, rs174546, and rs174550 in FADS1 were used as the genetic instruments of ALA/DPA, DHA, and AdrA, respectively. Rs99780 and rs472031 in FADS2 and FADS3 were used as the genetic instruments of LA and AA, respectively. Finally, rs174538 in C11orf10 was used as the genetic instrument of EPA.

In primary results (Tables 1, 2), we found no association of ALA [odds ratio (OR) = 1.09 per% changed, 95% confidence interval (CI) 0.67–1.78], EPA (OR = 0.94, 95% CI 0.88–1.01), DPA (OR = 0.95, 95% CI 0.88–1.02), and DHA (OR = 0.99, 95% CI 0.96–1.02) with the risk of SA using the IVW method. Moreover, for omega-6 PUFAs, no association between LA (OR = 0.98, 95% CI 0.96–1.01), AA (1.00, 95% CI 0.99–1.01), and AdrA (0.93, 95% CI 0.71–1.21) with the risk of SA was found. Similar results were found in sensitivity analyses using WM and MR-PRESSO and using SNPs with a *p*-value of < 5 × 10^–8^ (Supplementary Table 4). The MR-Egger intercept suggested no evidence for directional horizontal pleiotropy (all *p*-values > 0.05). Supplementary Figures 1,2 show the scatter plots of omega-3 and omega-6 PUFAs with SA in different methods. In sensitivity analysis, no associations of PUFAs with SA were found using the published GWAS from the FinnGen study (Supplementary Tables 5,6). There was no association of ALA and LA with SA found after adjustment for BMI (Supplementary Table 7).

**TABLE 1:** Mendelian randomization (MR) estimates of causality between plasma omega-3 polyunsaturated fatty acids (PUFAs) and sleep apnea (SA).

	Mendelian randomization method	No. of SNPs (mean F-statistic)	Odd ratio	95% Confidence interval	*P*-value	Cochran’s Q (I^2^)	MR-egger intercept (*P*-value)
α-linolenic acid (ALA)	IVW	6 (67.1)	1.09	0.67–1.78	0.738	8.14 (38.6%)	−0.003 (0.535)
	WM		1.21	0.79–1.84	0.385		
	MR Egger		1.42	0.52–3.87	0.526		
	MR-PRESSO		1.09	0.67–1.78	0.738		
Eicosapentaenoic acid (EPA)	IVW	17 (37.5)	0.94	0.88–1.01	0.095	18.8 (14.7%)	0.003 (0.467)
	WM		0.94	0.87–1.01	0.109		
	MR Egger		0.89	0.76–1.05	0.196		
	MR-PRESSO		0.94	0.88–1.01	0.095		
Docosapentaenoic acid (DPA)	IVW	12 (94.8)	0.95	0.88–1.02	0.169	11.7 (5.6%)	−0.005 (0.031)
	WM		0.96	0.88–1.06	0.447		
	MR Egger		1.06	0.94–1.20	0.375		
	MR-PRESSO		0.95	0.88–1.02	0.169		
Docosahexaenoic acid (DHA)	IVW	12 (34.4)	0.99	0.96–1.02	0.717	13.6 (19.4%)	0.003 (0.555)
	WM		0.99	0.95–1.03	0.691		
	MR Egger		0.96	0.86–1.08	0.519		
	MR-PRESSO		0.99	0.96–1.02	0.717		

IVW, inverse-variance weighted; WM, weighted median; MR-PRESSO, Mendelian randomization pleiotropy residual sum and outlier.

**TABLE 2:** Mendelian randomization (MR) estimates of causality between plasma omega-6 polyunsaturated fatty acids and sleep apnea.

	Mendelian randomization method	No. of SNPs (mean F-statistic)	Odd ratio	95% Confidence interval	*P*-value	Cochran’s Q (I^2^)	MR-egger intercept (*P*-value)
Linoleic acid (LA)	IVW	23 (42.3)	0.98	0.96–1.01	0.231	48.1 (54.2%)	0.000 (0.905)
	WM		0.99	0.96–1.01	0.320		
	MR Egger		0.99	0.93–1.05	0.671		
	MR-PRESSO		0.99	0.97–1.01	0.517		
Arachidonic acid (AA)	IVW	10 (40.8)	1.00	0.99–1.01	0.580	11.4 (21.2%)	0.003 (0.512)
	WM		1.00	0.99–1.02	0.398		
	MR Egger		0.99	0.96–1.02	0.938		
	MR-PRESSO		1.00	0.99–1.01	0.580		
Adrenic acid (AdrA)	IVW	5 (145.5)	0.93	0.71–1.21	0.580	8.2 (63.6%)	−0.009 (0.009)
	WM		0.94	0.81–1.09	0.399		
	MR Egger		0.98	0.61–1.57	0.938		
	MR-PRESSO		0.93	0.71–1.21	0.580		

IVW, inverse-variance weighted; WM, weighted median; MR-PRESSO, Mendelian randomization pleiotropy residual sum and outlier.

The single-locus MR analysis (Table 3) showed similar results to the primary results. A little genetic association of omega-3 and omega-6 PUFAs with SA was found, with the OR ranging from 0.94 to 1.21 (*p*-values from 0.144 to 0.937).

**TABLE 3:** Mendelian randomization (MR) estimates of causality between plasma polyunsaturated fatty acids and sleep apnea (FADS, C11orf10, and ELOVL gene loci).

Plasma polyunsaturated fatty acids		SNP (Gene)	No. of SNPs (Mean F-statistic)	Odd ratio	95% Confidence interval	*P*-value
Omega-3	α-linolenic acid (ALA)	rs174547 (FADS1)	1 (286.1)	1.21	0.76–1.90	0.419
	Eicosapentaenoic acid (EPA)	rs174538 (C11orf10)	1 (257.7)	0.94	0.86–1.02	0.144
	Docosapentaenoic acid (DPA)	rs174547 (FADS1)	1 (699.5)	0.96	0.87–1.06	0.420
	Docosahexaenoic acid (DHA)	rs174546 (FADS1)	1 (104.5)	0.98	0.93–1.04	0.478
Omega-6	Linoleic acid (LA)	rs99780 (FADS2)	1 (141.5)	1.02	0.97–1.07	0.509
	Arachidonic acid (AA)	rs472031 (FADS3)	1 (117.4)	1.00	0.97–1.02	0.937
	Adrenic acid (AdrA)	rs174550 (FADS1)	1 (635.1)	0.94	0.82–1.09	0.428

## Discussion

### Principal findings

Our MR analyses first showed no association between lifelong exposure to plasma omega-3 PUFAs (ALA, EPA, DPA, and EHA) and omega-6 PUFAs (LA, AA, and AdrA) with the risk of SA. Protective effects reported in previous observational studies might be explained by residual confounding.

### Comparison with other studies

Results of the present MR study did not support findings from previous observational studies showing that lower circulating omega-3 PUFAs (EPA and DHA) levels were associated with SA severity (12, 13, 30). A randomized, placebo-controlled trial showed that a daily intake of 600 mg omega-3 DHA supplements for 16 weeks improved sleep quality (less sleep disturbed breathing) (31). To date, no RCT assessed the effect of omega-6 PUFAs on SA (32), although better sleep quality was found after omega-6 PUFA supplementation in children with attention deficit hyperactivity disorder (ADHD) (33). Our study adds by clarifying that there is no causal effect of omega-3 and omega-6 PUFAs on SA, which is informative before an RCT and can make the most effective use of scarce resources.

### Possible mechanisms

The underlying mechanisms explaining associations between PUFAs and SA are far from clear. One of the proposed mechanisms was an inflammatory response to obstructive sleep apnea (OSA), i.e., mediating by cytokines, such as tumor necrosis factor (TNF-α) (15) and interleukin 6 (IL-6) (34). Animal and human studies have shown that the production of cytokines can be reduced by omega-3 PUFAs (35–37). However, the causal association between cytokines and SA was not evident. Therefore, whether the associations of PUFAs with SA are causal, a reflection of the underlying comorbidities, or merely due to chance, is yet to be confirmed, although this mechanism pathway seems reasonable.

### Strengths and limitations

To our knowledge, our study is the first MR study examining the effect of omega-3 and omega-6 PUFAs on the risk of SA. The strengths of the present study included the use of MR to minimize residual confounding and reverse causality in traditional observational studies, and genetic validation of the wide range of PUFAs. Nevertheless, several limitations exist. MR is based on three stringent assumptions, i.e., the genetic instruments are strongly associated with the exposure; no confounders for the associations between the genetic instruments and the outcome; and the genetic instruments are not linked with the outcome other than *via* the exposure (no pleiotropy). To satisfy these assumptions, we only selected SNPs strongly associated with PUFAs reaching suggestive genome-wide significance, and replicated our findings using the most functionally related SNP in chromosome 11 or 6, including the well-established gene FADS (18). Population stratification might be a confounder in MR studies. However, we only used studies involving people of European descent, with genomic control. Confounding due to population stratification should be minimized. Regarding the potential pleiotropic effects, we conducted sensitivity analyses using multiple methods (i.e., WM, MR-PRESSO, and MVMR) to assess pleiotropy and found no evidence of a pleiotropic effect. Second, the estimates might be biased toward the observational associations if the exposure and outcome data came from the same sample (38). However, the sample of PUFAs GWAS had no overlap with the UK Biobank. Third, the effects of endogenous PUFAs may not be exactly the same as those of PUFAs from dietary intake. However, the essential fatty acids in omega-3 and omega-6 PUFA families, i.e., LA and ALA, cannot be synthesized directly in the human body (39). Serum LA levels are associated with dietary intake of LA (40). Moreover, the use of genetically predicted plasma PUFAs can eliminate measurement error, since the observational studies use one snapshot of measurement rather than lifetime exposure (41). Fourth, since cases of SA and snoring were identified by clinic diagnosis or self-report, misclassification was inevitable in the current study. However, the genetic correlation analyses from our upstream GWAS of SA (22) suggested different diagnostic criteria (i.e., by clinic diagnosis or self-report) have a comparable genetic architecture. After all, ascertaining SA cases using objective measures is difficult for GWAS with a large sample size. Fifth, the possible non-linear associations of PUFAs with SA could not be examined in the present study using summary-level statistics. Further studies using individual-level data are warranted to explore the potential non-linear patterns. Sixth, we could not distinguish the effects of central sleep apnea (CSA) and obstructive sleep apnea (OSA) and thus only examined SA in general, although SA likely represents the effect of OSA given the much higher prevalence of OSA (42).

### Conclusion and public health implications

We first found no genetic evidence supporting the causal role of omega-3 and omega-6 PUFAs in the risk of SA. From a public health perspective, our findings refute the notion that consumption of foods rich in PUFAs or the use of PUFAs supplementation can reduce the risk of SA. Further MR studies, especially studies from other populations, providing more objective-diagnosed cases of SA and, ideally, using additional variants as genetic instruments are warranted to replicate the results.

## Data availability statement

The exposure data are available on request after approval by AC and MR. The outcome data underlying this article are available in these articles and in their online Supplementary material.

## Author contributions

JW, AC, MR, and LX made substantial contributions to the conception and design and interpretation of data. JW and LX analyzed the data and drafted the article. YH, HY, ZL, AC, and MR revised it critically for important intellectual content. LX was guarantor. All authors gave their final approval for the manuscript.

## Footnotes

1
https://shiny.cnsgenomics.com/mRnd/
