# Supplementary material for: Plasma polyunsaturated fatty acid concentrations and sleep apnea risk: A two-sample Mendelian randomization study
Source: Front Nutr. 2022 Aug 18;9:956900. doi: 10.3389/fnut.2022.956900 (PMC9433775; doi:10.3389/fnut.2022.956900)
Supplement: Supplementary file 1 [file Data_Sheet_1.pdf]

Plasma polyunsaturated fatty acid concentrations and sleep apnea: a two-sample Mendelian randomization study

(Supplementary tables and figures)

Content

Supplementary methods

Table S1. Study details for the GWAS of polyunsaturated fatty acids. 3
Table S2. Summary information on the omega-3 polyunsaturated fatty acids genetic instruments used in the Mendelian randomization analyses. 4
Table S3. Summary information on the omega-6 polyunsaturated fatty acids genetic instruments used in the Mendelian randomization analyses. 6
Table S4. Mendelian randomization (MR) estimates of causality between plasma polyunsaturated fatty acids (PUFAs) and sleep apnea (P value<5×10^{-8}). 8
Table S5. Mendelian randomization (MR) estimates of causality between plasma omega-3 polyunsaturated fatty acids and sleep apnea using published GWAS from FinnGen Study. 9
Table S6. Mendelian randomization (MR) estimates of causality between plasma omega-6 polyunsaturated fatty acids and sleep apnea using published GWAS from FinnGen Study. 10
Table S7. Mendelian randomization (MR) estimates of causality between plasma polyunsaturated fatty acids and sleep apnea after adjustment for body mass index (BMI). 11

Figure S1. Methods comparison plots of omega-3 polyunsaturated fatty acids on sleep apnea in two-sample MR 12
Figure S2. Methods comparison plots of omega-6 polyunsaturated fatty acids on sleep apnea in two-sample MR 13

Supplementary methods

Genetic associations with sleep apnea and snoring

The summary-level statistics are obtained from the meta-analysis of GWAS from five cohort: UK-Biobank, FinnGen, Canadian Longitudinal Study of Aging, Australian genetics of depression study, and Partners Biobank (USA). Details of the five cohort is: (1). **UK-Biobank.** The UK-Biobank genotyping has been extensively described elsewhere[1]. For sleep apnea, phenotypic data from three different sources were used to define cases: (i) ICD sleep apnea diagnosis code (code G47.3 in the 41280 data field), (ii) self-reported sleep apnea (code 1123 in the 20002 data field) and data from GP records (codes corresponding to "G473" within the UK -Biobank primary care data). (2). **FinnGen**. Sleep apnea analysis comprised 9,096 cases and 110,963 controls. The diagnosis of OSA was based on ICD-codes (ICD-10: G47.3, ICD-9: 3472A), which were obtained from the Finnish National Hospital Discharge Registry and the Causes of Death Registry[3]. (3). **Canadian Longitudinal Study of Aging (CLSA)**. CLSA collected data at baseline from 2011 until 2015 and data follow-up was from 2015 until 2018. To identify individuals with sleep apnea, following recorded data categories "SNO_STOPBREATH_MCQ" and "SNO_STOPBREATH_COF1" from baseline and follow-up respectively was used. This data was collected through questionnaires, the question label was 'stop breathing in sleep' to which possible answers were: "Yes", "No' or "Don't Know". Individuals who answered "Yes" were defined as cases, those answering "No" were considered controls. Individuals who did not answer or answered "Don't Know" were excluded. (4). **Australian genetics of depression study (AGDS). AGDS** comprises ~20,000 participants of which more than 18,000 have been genotyped[6]. Phenotypic data for sleep apnea was obtained through self-reported questionnaires. Briefly, the item to ascertain sleep apnea was: "During the last month, on how many nights or days per week have you had or been told that your breathing stops or you choke or struggle for breath". Participants that responded "Never" were coded as controls. (5). **Partners Health Care Biobank**. Data for over 35,000 volunteers is available[7]. Sleep apnea cases were defined based on a diagnosis available on electronic health records. Controls were participants not showing a sleep apnea ICD code.

Table S1. Study details for the GWAS of polyunsaturated fatty acids.

	Traits	Year	Studies	Population	Pubmed
**Exposures**	Omega-3 polyunsaturated fatty acids	2011	ARIC Study, CARDIA Study, CHS Study, InCHIANTI study, and an ancillary study to MESA	8866 individuals of European ancestry	21829377
	Omega-6 polyunsaturated fatty acids	2014	ARIC Study, CARDIA Study, CHS Study, InCHIANTI study, and an ancillary study to MESA	8631 individuals of European ancestry	24823311
	Meta of omega-3 and omega-6 polyunsaturated fatty acids	2016	EGCUT, ERF, FTC, FR97, COROGENE, GenMets, HBCS, KORA, LLS,NTR NFBC 1966, PredictCVD, PROTE,YFS	24,925 individuals of European ancestry	27005778
**Outcomes**	Sleep apnea	2022	UK biobank, CLSA, AGDS, FinnGen; USA (Partner's Healthcare Biobank)	523,366 individuals of European ancestry	-
	Sleep apnea (In sensitivity analysis)	2021	FinnGen	217,955 individuals of European ancestry	33243845

ARIC, Atherosclerosis Risk in Communities. CARDIA, Coronary Artery Risk Development in Young Adults, CHS, Cardiovascular Health Study. InCHIANTI, Invecchiare in Chianti. COROGENE, Genetic Predisposition of Coronary Heart Disease in Patients Verified with Coronary Angiogram; EGCUT, Estonian Genome Center of University of Tartu Cohort; ERF, Erasmus Rucphen Family Study; FR97, a subsample of FINRISK 1997; FTC, Finnish Twin Cohort; GenMets, Genetics of METabolic Syndrome; HBCS, Helsinki Birth Cohort Study; KORA, Cooperative Health Research in the Region of Augsburg; LLS, Leiden Longevity Study; N, number of individuals with both genotype and metabolite traits analysed; NFBC 1966, Northern Finland Birth Cohort 1966; NTR, Netherlands Twin Register; PredictCVD, FINRISK subsample of incident cardiovascular cases and controls; PROTE, EGCUT sub-cohort; YFS, The Cardiovascular Risk in Young Finns Study; UKB: UK Biobank; CLSA: Canadian Longitudinal Study of Aging; AGDS: Australian Genetics of Depression Study; FinnGen: Finland genetic research

Table S2. Summary information on the omega-3 polyunsaturated fatty acids genetic instruments used in the Mendelian randomization analyses.

	SNP	Chr	Position	Beta	SE	F_statistic	Effect_allele	P_value	Gene
ALA	rs174547	11	61327359	-0.02	0.00	286.13	T	3.47E-64	FADS1
	rs16832011	2	136261769	0.02	0.00	26.16	A	3.15E-07	LCT
	rs7200543	16	15037471	0.00	0.00	24.17	A	8.80E-07	PDXDC1
	rs367543	8	9071558	0.01	0.00	23.44	T	1.29E-06	PPP1R3B
	rs6872	1	26017261	0.01	0.00	21.83	A	2.98E-06	SEPN1
	rs4135168	9	112056706	0.00	0.00	20.86	T	4.95E-06	TXN
EPA	rs174538	11	61316657	-0.08	0.01	257.73	A	5.37E-58	C11orf10
	rs1145652	5	164696665	0.04	0.01	28.71	A	8.39E-08	MAT2B
	rs1514178	1	60978057	0.07	0.01	26.77	T	2.29E-07	NFIA
	rs2585617	6	69394508	-0.03	0.01	24.93	A	5.95E-07	BAI3
	rs12459897	19	36288618	-0.09	0.02	24.32	T	8.16E-07	TSHZ3
	rs10847425	12	126700427	0.03	0.01	23.91	A	1.01E-06	SLC15A4
	rs5749970	22	33560810	-0.06	0.01	23.12	T	1.52E-06	ISX
	rs3007728	1	18666828	-0.06	0.01	22.94	T	1.67E-06	KLHDC7A
	rs4499314	18	26165113	-0.08	0.02	22.71	T	1.88E-06	DSC3
	rs7480288	11	121230316	0.04	0.01	22.66	T	1.93E-06	SORL1
	rs10512289	9	103550279	0.08	0.02	22.35	T	2.27E-06	GRIN3A
	rs7076844	10	19866244	-0.03	0.01	22.24	A	2.40E-06	PLXDC2
	rs4691309	4	157340664	-0.05	0.01	22.11	A	2.57E-06	CTSO
	rs7207094	17	29749782	-0.03	0.01	22.00	A	2.72E-06	CCL1
	rs17047227	1	216265018	-0.07	0.02	21.08	T	4.40E-06	SPATA17
	rs2542749	18	26385123	0.04	0.01	21.06	A	4.45E-06	DSC3
	rs6450352	5	55144489	0.04	0.01	20.93	A	4.77E-06	DDX4
DPA	rs174547	11	61327359	0.07	0.00	699.53	T	3.79E-154	FADS1
	rs3734398	6	11090959	-0.04	0.00	192.38	T	9.61E-44	ELOVL2
	rs780094	2	27594741	0.02	0.00	33.04	T	9.04E-09	GCKR
	rs7435	21	44228766	-0.02	0.00	26.68	A	2.40E-07	AGPAT3
	rs9586179	13	102858088	0.05	0.01	25.79	T	3.81E-07	SLC10A2
	rs12587311	14	28186446	-0.01	0.00	25.15	T	5.31E-07	FOXG1
	rs6844153	4	26553412	0.02	0.00	24.93	T	5.94E-07	STIM2
	rs9357021	6	11014140	-0.02	0.00	24.86	A	6.18E-07	SYCP2L
	rs951500	4	83878052	-0.04	0.01	21.44	T	3.65E-06	SCD5
	rs2642438	1	219036651	-0.02	0.00	21.17	A	4.20E-06	MOSC1
	rs9313625	5	172622830	-0.01	0.00	21.17	A	4.20E-06	NKX2-5
	rs4706778	6	80121944	-0.02	0.00	21.04	A	4.50E-06	HMGN3
DHA	rs174546	11	61569830	-0.13	0.01	104.54	T	4.81E-24	FADS1
	rs145717049	19	19130096	-0.2	0.03	37.78	T	1.21E-09	SUGP2
	rs2281591	6	10990493	0.11	0.02	35.57	A	3.66E-09	ELOVL2
	rs11604424	11	116651115	-0.08	0.01	34.06	T	7.84E-09	ZPR1
	rs10468017	15	58678512	0.07	0.01	28.59	T	1.23E-07	ALDH1A2

rs3020043	11	70985372	0.09	0.02	27.95	T	1.71E-07	SHANK2
rs11227881	11	67462549	-0.07	0.01	27.34	A	2.32E-07	RPL37P2
rs2141922	2	11016393	0.07	0.01	26.19	A	4.16E-07	LINC01954
rs9308453	1	9503644	0.06	0.01	24.14	A	1.17E-06	RNA5SP40
rs6744096	2	80632556	0.09	0.02	22.41	T	2.84E-06	CTNNA2
rs10160784	11	75456055	0.07	0.01	22.07	T	3.37E-06	RN7SL786P
rs1260326	2	27730940	0.06	0.01	21.94	T	3.60E-06	GCKR

SE: standard error. Note: BETA and SE are responsible for the effect allele.

Table S3. Summary information on the omega-6 polyunsaturated fatty acids genetic instruments used in the Mendelian randomization analyses.

	SNP	Chr	Position	BATE	SE	F_statistic	Effect_allele	P_value	Gene
Linoleic acid (LA)	rs99780	11	61596633	0.15	0.01	141.52	T	3.35E-32	FADS2
	rs7412	19	45412079	-0.30	0.03	109.07	T	3.40E-25	APOE
	rs1800588	15	58723675	0.13	0.01	79.78	T	7.41E-19	LIPC
	rs769449	19	45410002	0.14	0.02	70.40	A	8.04E-17	APOE
	rs79225634	5	74619639	0.10	0.01	55.16	T	1.66E-13	CTD-2235C13.2
	rs174418	15	58687603	0.09	0.01	47.12	T	9.42E-12	ALDH1A2
	rs1260326	2	27730940	0.08	0.01	41.94	T	1.28E-10	GCKR
	rs4296389	2	21142994	-0.08	0.01	38.76	T	6.37E-10	RP11-116D2.1
	rs144064722	4	73406173	-0.23	0.04	33.90	A	7.45E-09	ADAMTS3
	rs76366838	19	45399896	0.29	0.05	33.51	A	9.06E-09	TOMM40
	rs144723570	21	45244466	-0.33	0.06	30.89	T	3.42E-08	-
	rs9804646	11	116665079	-0.11	0.02	30.56	T	4.05E-08	APOA5
	rs17699030	19	11330942	0.17	0.03	28.61	A	1.09E-07	DOCK6
	rs629301	1	109818306	0.08	0.01	26.89	T	2.62E-07	CELSR2
	rs76246956	4	74783906	0.20	0.04	25.65	A	4.93E-07	RP11-576N17.4
	rs76488675	1	56885874	0.14	0.03	23.49	A	1.50E-06	LINC01767
	rs6558405	8	144991176	-0.06	0.01	23.02	T	1.90E-06	PLEC
	rs10410208	19	46078485	0.07	0.01	22.47	T	2.52E-06	OPA3
	rs6543521	2	240226642	0.08	0.02	22.32	T	2.72E-06	HDAC4
	rs116285936	3	49195952	-0.38	0.08	22.27	T	2.80E-06	CCDC71
	rs11878174	19	45723379	-0.07	0.01	22.15	T	2.97E-06	EXOC3L2
	rs1547615	6	116985976	-0.07	0.01	21.73	T	3.68E-06	ZUFSP
	rs1848922	2	21471603	-0.07	0.02	21.31	T	4.56E-06	AC067959.1
Arachidonic acid (AA)	rs472031	11	61394996	0.51	0.05	117.41	A	2.34E-27	FADS3
	rs760306	11	61480868	-0.28	0.04	62.69	T	2.42E-15	BEST1
	rs12285167	11	61248615	-0.28	0.04	50.75	A	1.05E-12	DAGLA
	rs16829840	3	120630648	0.46	0.08	31.04	T	2.53E-08	TMEM39A
	rs3741259	11	61038926	-0.31	0.06	29.92	T	4.50E-08	SYT7
	rs17663676	11	61957578	-0.47	0.09	29.91	T	4.52E-08	AHNAK
	rs9394931	6	42981223	-0.15	0.03	22.33	T	2.30E-06	PTCRA
	rs12209128	6	47990507	-0.15	0.03	22.06	T	2.64E-06	C6orf138
	rs274557	5	131749103	0.14	0.03	21.52	T	3.51E-06	SLC22A5
	rs12471016	2	76058497	-0.25	0.05	21.34	T	3.84E-06	C2orf3
Adrenic acid (AdrA)	rs174550	11	61328054	0.05	0.002	635.05	T	3.98E-140	FADS1
	rs10761785	10	64988772	0.01	0.002	24.75	T	6.51E-07	REEP3
	rs1962772	8	17415157	0.01	0.002	24.14	T	8.96E-07	SLC7A2

rs4083482	11	35308215	-0.03	0.006	22.94	T	1.67E-06	SLC1A2
rs3134950	6	32235455	-0.01	0.002	20.6	A	5.72E-06	PPT2

SE: standard error. Note: BETA and SE are responsible for the effect allele.

Table S4. Mendelian randomization (MR) estimates of causality between plasma polyunsaturated fatty acids (PUFAs) and sleep apnea (P value<5×10^{-8}).

	**PUFAs**	**Mendelian Randomization Method**	**No. of SNPs (mean F-statistic)**	**Odd ratio**	**95% Confidence Interval**	**P-value**
Omega-3	α-linolenic acid (ALA)	IVW	1 (286.1)	1.21	0.76-1.90	0.419
	Eicosapentaenoic acid (EPA)	IVW	1 (257.7)	0.94	0.86-1.02	0.144
	Docosapentaenoic acid (DPA)	IVW	3 (308.3)	0.98	0.89-1.09	0.720
	Docosahexaenoic acid (DHA)	IVW	4 (52.9)	0.97	0.93-1.01	0.123
Omega-6	Linoleic acid (LA)	IVW	10 (61.2)	0.98	0.95-1.02	0.397
	Arachidonic acid (AA)	IVW	2 (41.5)	0.97	0.93-1.02	0.271
	Adrenic acid (AdrA)	IVW	1 (635.1)	0.94	0.82-1.09	0.428

Note: IVW: inverse-variance weighted.

Table S5. Mendelian randomization (MR) estimates of causality between plasma omega-3 polyunsaturated fatty acids and sleep apnea using published GWAS from FinnGen Study.

	**Mendelian Randomization Method**	**No. of SNPs (mean F-statistic)**	**Odd ratio**	**95% Confidence Interval**	**P-value**	**Cochran's Q (I^2)**	**MR-Egger intercept (p-value)**
α-linolenic acid (ALA)	IVW	6 (60.8)	3.19	0.51-19.9	0.213	11.2 (55.6%)	-0.001 (0.974)
	WM		2.66	0.66-10.7	0.169		
	MR Egger		3.37	0.07-165.8	0.573		
	MR-PRESSO		3.19	0.51-19.9	0.213		
Eicosapentaenoic acid (EPA)	IVW	18 (33.9)	0.85	0.64-1.03	0.104	23.4 (1.6%)	-0.005 (0.673)
	WM		0.83	0.64-1.09	0.176		
	MR Egger		0.86	0.53-1.37	0.529		
	MR-PRESSO		0.85	0.64-1.03	0.104		
Docosapentaenoic acid (DPA)	IVW	12 (84.8)	0.86	0.68-1.09	0.218	14.2 (8.7%)	0.007 (0.268)
	WM		0.84	0.64-1.11	0.221		
	MR Egger		0.78	0.51-1.19	0.271		
	MR-PRESSO		0.86	0.68-1.09	0.218		
Docosahexaenoic acid (DHA)	IVW	15 (34.3)	0.93	0.85-1.01	0.096	12.4 (0.198)	0.008 (0.472)
	WM		0.91	0.82-1.02	0.113		
	MR Egger		0.82	0.62-1.09	0.197		
	MR-PRESSO		0.93	0.85-1.01	0.096		

Note: IVW: inverse-variance weighted; WM: weighted median; MR-PRESSO: Mendelian randomization pleiotropy residual sum and outlier.

Table S6. Mendelian randomization (MR) estimates of causality between plasma omega-6 polyunsaturated fatty acids and sleep apnea using published GWAS from FinnGen Study.

	**Mendelian Randomization Method**	**No. of SNPs (Mean F-statistic)**	**Odd ratio**	**95% Confidence Interval**	**P-value**	**Cochran's Q (I^2)**	**MR-Egger intercept (p-value)**
Linoleic acid (LA)	IVW	27 (44.7)	0.98	0.95-1.03	0.523	32.3 (7.0%)	0.003 (0.602)
	WM		0.97	0.90-1.05	0.486		
	MR Egger		0.96	0.87-1.07	0.503		
	MR-PRESSO		0.98	0.95-1.03	0.523		
Arachidonic acid (AA)	IVW	10 (41.4)	0.98	0.93-1.03	0.337	22.1 (50.2%)	0.019 (0.234)
	WM		0.97	0.93-1.03	0.382		
	MR Egger		0.93	0.82-1.07	0.338		
	MR-PRESSO		0.98	0.93-1.03	0.337		
Adrenic acid (AdrA)	IVW	6 (125.4)	0.68	0.39-1.20	0.183	7.13 (29.9%)	0.002 (0.873)
	WM		0.74	0.47-1.16	0.184		
	MR Egger		0.65	0.24-1.79	0.467		
	MR-PRESSO		0.68	0.39-1.20	0.183		

Note: IVW: inverse-variance weighted; WM: weighted median; MR-PRESSO: Mendelian randomization pleiotropy residual sum and outlier.

Table S7. Mendelian randomization (MR) estimates of causality between plasma polyunsaturated fatty acids and sleep apnea after adjustment for body mass index (BMI).

**Plasma polyunsaturated fatty acids**		**Odd ratio**	**95% Confidence Interval**	**P-value**
Omega-3	α-linolenic acid (ALA)	0.93	0.50-1.73	0.812
Omega-6	Linoleic acid (LA)	0.99	0.94-1.04	0.630

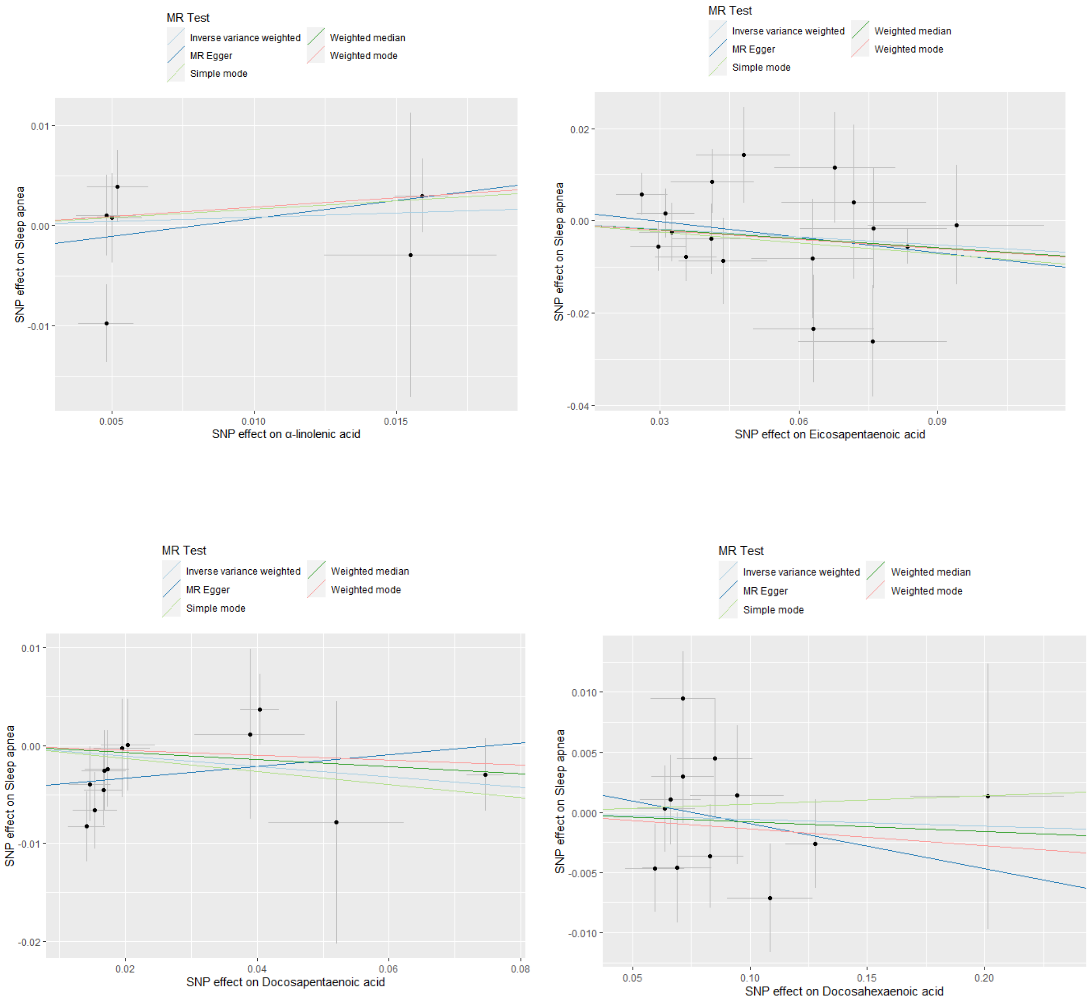

Figure S1. Methods comparison plots of omega-3 polyunsaturated fatty acids on sleep apnea in two-sample MR

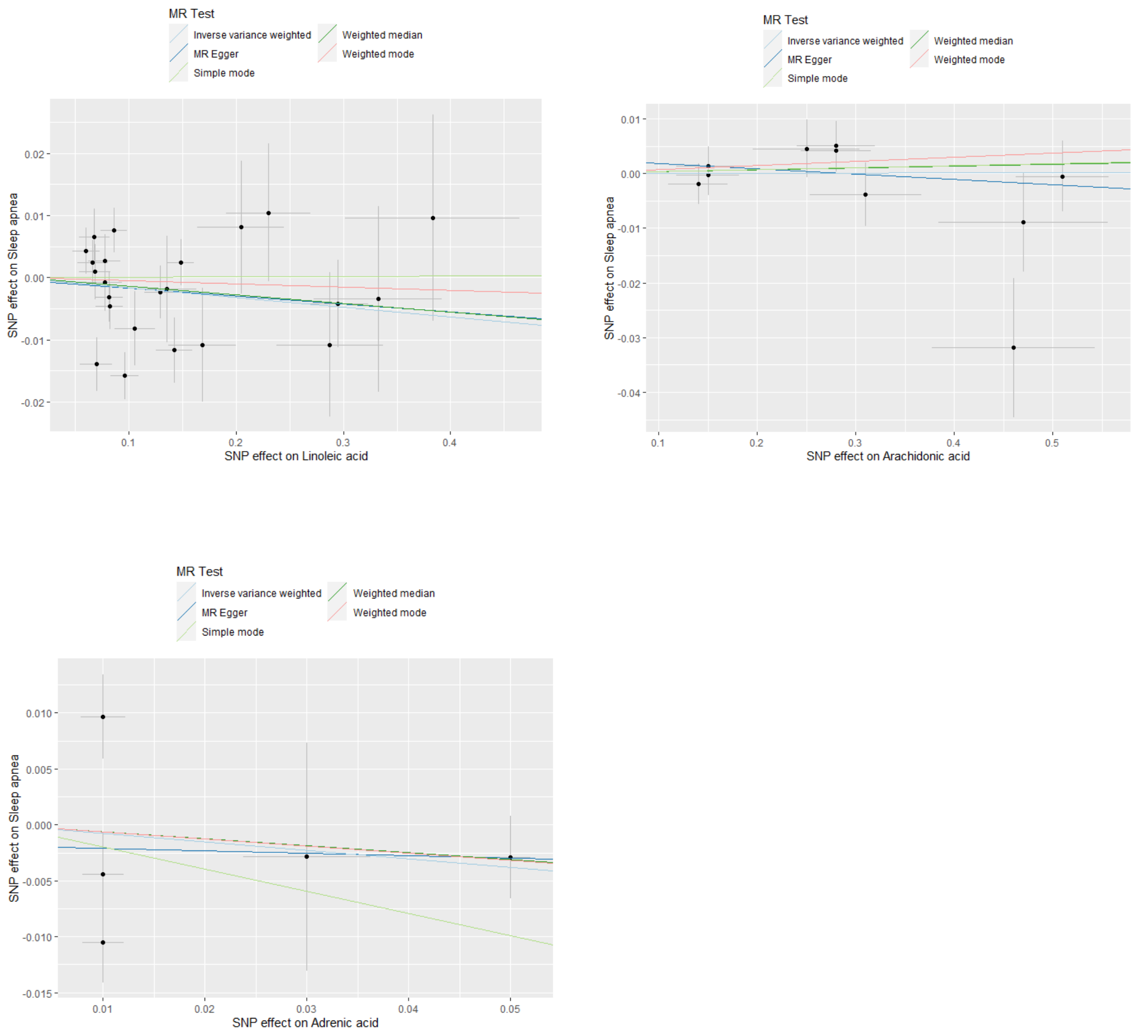

Figure S2. Methods comparison plots of omega-6 polyunsaturated fatty acids on sleep apnea in two-sample MR

Reference:

1. Benjafield AV, Ayas NT, Eastwood PR, Heinzer R, Ip MSM, Morrell MJ, Nunez CM, Patel SR, Penzel T, Pépin JL *et al*: **Estimation of the global prevalence and burden of obstructive sleep apnoea: a literature-based analysis**. *Lancet Respir Med* 2019, **7**(8):687-698.
2. Wang X, Ouyang Y, Wang Z, Zhao G, Liu L, Bi Y: **Obstructive sleep apnea and risk of cardiovascular disease and all-cause mortality: a meta-analysis of prospective cohort studies**. *Int J Cardiol* 2013, **169**(3):207-214.
3. Huang T, Lin BM, Stampfer MJ, Tworoger SS, Hu FB, Redline S: **A Population-Based Study of the Bidirectional Association Between Obstructive Sleep Apnea and Type 2 Diabetes in Three Prospective U.S. Cohorts**. *Diabetes Care* 2018, **41**(10):2111-2119.
4. Emamian F, Khazaie H, Tahmasian M, Leschziner GD, Morrell MJ, Hsiung G-YR, Rosenzweig I, Sepehry AA: **The Association Between Obstructive Sleep Apnea and Alzheimer's Disease: A Meta-Analysis Perspective**. *Front Aging Neurosci* 2016, **8**:78-78.
5. Shamsuzzaman AS, Winnicki M, Lanfranchi P, Wolk R, Kara T, Accurso V, Somers VK: **Elevated C-reactive protein in patients with obstructive sleep apnea**. *Circulation* 2002, **105**(21):2462-2464.
6. Cao Y, Song Y, Ning P, Zhang L, Wu S, Quan J, Li Q: **Association between tumor necrosis factor alpha and obstructive sleep apnea in adults: a meta-analysis update**. *BMC Pulmonary Medicine* 2020, **20**(1):215.
7. Zhao JV, Schooling CM: **Effect of linoleic acid on ischemic heart disease and its risk factors: a Mendelian randomization study**. *BMC Med* 2019, **17**(1):61.
8. Coelho OGL, da Silva BP, Rocha D, Lopes LL, Alfenas RCG: **Polyunsaturated fatty acids and type 2 diabetes: Impact on the glycemic control mechanism**. *Crit Rev Food Sci Nutr* 2017, **57**(17):3614-3619.
9. Zhao JV, Schooling CM: **The role of linoleic acid in asthma and inflammatory markers: a Mendelian randomization study**. *Am J Clin Nutr* 2019, **110**(3):685-690.
10. Alzoubi MR, Aldomi Al-Domi H: **Could omega-3 fatty acids a therapeutic treatment of the immune-metabolic consequence of intermittent hypoxia in obstructive sleep apnea?** *Diabetes Metab Syndr* 2017, **11**(4):297-304.
11. Tricon S, Burdge GC, Kew S, Banerjee T, Russell JJ, Grimble RF, Williams CM, Calder PC, Yaqoob P: **Effects of cis-9,trans-11 and trans-10,cis-12 conjugated linoleic acid on immune cell function in healthy humans**. *Am J Clin Nutr* 2004, **80**(6):1626-1633.
12. Ladesich JB, Pottala JV, Romaker A, Harris WS: **Membrane level of omega-3 docosahexaenoic acid is associated with severity of obstructive sleep apnea**. *J Clin Sleep Med* 2011, **7**(4):391-396.
13. Tittus J, Huber MT, Storck K, Köhler A, Köhler JM, von Arnim T, von Schacky C: **Omega-3 Index and Obstructive Sleep Apnea: A Cross-Sectional Study**. *J Clin Sleep Med* 2017, **13**(10):1131-1136.
14. Cicero AF, Ferroni A, Ertek S: **Tolerability and safety of commonly used dietary supplements and nutraceuticals with lipid-lowering effects**. *Expert opinion on drug safety* 2012, **11**(5):753-766.
15. Scorza FA, Cavalheiro EA, Scorza CA, Galduróz JC, Tufik S, Andersen ML: **Sleep Apnea and Inflammation - Getting a Good Night's Sleep with Omega-3 Supplementation**.

Front Neurol 2013, **4**:193.
16. Yuan S, Larsson SC: **Association of genetic variants related to plasma fatty acids with type 2 diabetes mellitus and glycaemic traits: a Mendelian randomisation study**. *Diabetologia* 2020, **63**(1):116-123.
17. Guan W, Steffen BT, Lemaitre RN, Wu JHY, Tanaka T, Manichaikul A, Foy M, Rich SS, Wang L, Nettleton JA *et al*: **Genome-wide association study of plasma N6 polyunsaturated fatty acids within the cohorts for heart and aging research in genomic epidemiology consortium**. *Circ Cardiovasc Genet* 2014, **7**(3):321-331.
18. Kettunen J, Demirkan A, Würtz P, Draisma HHM, Haller T, Rawal R, Vaarhorst A, Kangas AJ, Lyytikäinen L-P, Pirinen M *et al*: **Genome-wide study for circulating metabolites identifies 62 loci and reveals novel systemic effects of LPA**. *Nature Communications* 2016, **7**(1):11122.
19. Lemaitre RN, Tanaka T, Tang W, Manichaikul A, Foy M, Kabagambe EK, Nettleton JA, King IB, Weng LC, Bhattacharya S *et al*: **Genetic loci associated with plasma phospholipid n-3 fatty acids: a meta-analysis of genome-wide association studies from the CHARGE Consortium**. *PLoS Genet* 2011, **7**(7):e1002193.
20. Campos AI, Ingold N, Huang Y, Mitchell BL, Kho P-F, Han X, García-Marín LM, Ong J-S, Team aR, Law MH *et al*: **Multi-trait genome-wide association study identifies new loci associated with sleep apnoea risk**. *medRxiv* 2022:2020.2009.2029.20199893.
21. Turley P, Walters RK, Maghzian O, Okbay A, Lee JJ, Fontana MA, Nguyen-Viet TA, Wedow R, Zacher M, Furlotte NA *et al*: **Multi-trait analysis of genome-wide association summary statistics using MTAG**. *Nat Genet* 2018, **50**(2):229-237.
22. Strausz S, Ruotsalainen S, Ollila HM, Karjalainen J, Kiiskinen T, Reeve M, Kurki M, Mars N, Havulinna AS, Luonsi E *et al*: **Genetic analysis of obstructive sleep apnoea discovers a strong association with cardiometabolic health**. *Eur Respir J* 2020.
23. Bowden J, Del Greco MF, Minelli C, Davey Smith G, Sheehan NA, Thompson JR: **Assessing the suitability of summary data for two-sample Mendelian randomization analyses using MR-Egger regression: the role of the I2 statistic**. *Int J Epidemiol* 2016, **45**(6):1961-1974.
24. Richardson TG, Sanderson E, Palmer TM, Ala-Korpela M, Ference BA, Davey Smith G, Holmes MV: **Evaluating the relationship between circulating lipoprotein lipids and apolipoproteins with risk of coronary heart disease: A multivariable Mendelian randomisation analysis**. *PLoS Med* 2020, **17**(3):e1003062.
25. Verbanck M, Chen CY, Neale B, Do R: **Detection of widespread horizontal pleiotropy in causal relationships inferred from Mendelian randomization between complex traits and diseases**. *Nat Genet* 2018, **50**(5):693-698.
26. Freeman G, Cowling BJ, Schooling CM: **Power and sample size calculations for Mendelian randomization studies using one genetic instrument**. *Int J Epidemiol* 2013, **42**(4):1157-1163.
27. Nakabayashi K, Jujo K, Saito K, Oka T, Hagiwara N: **Evaluation of the association between sleep apnea and polyunsaturated fatty acids profiles in patients after percutaneous coronary intervention**. *Heart Vessels* 2017, **32**(11):1296-1303.
28. Montgomery P, Burton JR, Sewell RP, Spreckelsen TF, Richardson AJ: **Fatty acids and sleep in UK children: subjective and pilot objective sleep results from the DOLAB study--a**

randomized controlled trial. *J Sleep Res* 2014, **23**(4):364-388.

29. Zhao M, Tuo H, Wang S, Zhao L: **The Effects of Dietary Nutrition on Sleep and Sleep Disorders**. *Mediators of Inflammation* 2020, **2020**:3142874.
30. Yehuda S, Rabinovitz-Shenkar S, Carasso RL: **Effects of essential fatty acids in iron deficient and sleep-disturbed attention deficit hyperactivity disorder (ADHD) children**. *Eur J Clin Nutr* 2011, **65**(10):1167-1169.
31. Ünüvar Doğan F, Yosunkaya Ş, Kuzu Okur H, Can Ü: **Relationships between Obstructive Sleep Apnea Syndrome, Continuous Positive Airway Pressure Treatment, and Inflammatory Cytokines**. *Sleep Disorders* 2014, **2014**:518920.
32. Meydani SN: **Effect of (n-3) polyunsaturated fatty acidson cytokine production and their biologic function**. *Nutrition* 1996, **12**(1, Supplement):S8-S14.
33. Kiecolt-Glaser JK, Belury MA, Andridge R, Malarkey WB, Glaser R: **Omega-3 supplementation lowers inflammation and anxiety in medical students: a randomized controlled trial**. *Brain Behav Immun* 2011, **25**(8):1725-1734.
34. Madison AA, Belury MA, Andridge R, Renna ME, Rosie Shrout M, Malarkey WB, Lin J, Epel ES, Kiecolt-Glaser JK: **Omega-3 supplementation and stress reactivity of cellular aging biomarkers: an ancillary substudy of a randomized, controlled trial in midlife adults**. *Mol Psychiatry* 2021, **26**(7):3034-3042.
35. Burgess S, Davies NM, Thompson SG: **Bias due to participant overlap in two-sample Mendelian randomization**. *Genetic epidemiology* 2016, **40**(7):597-608.
36. Nagy K, Tiuca I-D: **Importance of Fatty Acids in Physiopathology of Human Body**. In., edn.; 2017.
37. Ramsden CE, Zamora D, Leelarthaepin B, Majchrzak-Hong SF, Faurot KR, Suchindran CM, Ringel A, Davis JM, Hibbeln JR: **Use of dietary linoleic acid for secondary prevention of coronary heart disease and death: evaluation of recovered data from the Sydney Diet Heart Study and updated meta-analysis**. *Bmj* 2013, **346**:e8707.
38. Javaheri S, Barbe F, Campos-Rodriguez F, Dempsey JA, Khayat R, Javaheri S, Malhotra A, Martinez-Garcia MA, Mehra R, Pack AI *et al*: **Sleep Apnea: Types, Mechanisms, and Clinical Cardiovascular Consequences**. *J Am Coll Cardiol* 2017, **69**(7):841-858.
